# Modification Strategies of High-Energy Li-Rich Mn-Based Cathodes for Li-Ion Batteries: A Review

**DOI:** 10.3390/molecules29051064

**Published:** 2024-02-29

**Authors:** Zhenjie Xi, Qing Sun, Jing Li, Ying Qiao, Guanghui Min, Lijie Ci

**Affiliations:** 1 State Key Laboratory of Advanced Welding and Joining, School of Materials Science and Engineering, Harbin Institute of Technology (Shenzhen), Shenzhen 518055, China; 2 Key Laboratory for Liquid-Solid Structural Evolution & Processing of Materials (Ministry of Education), School of Materials Science and Engineering, Shandong University, Jinan 250061, China

**Keywords:** lithium-ion batteries, lithium-rich manganese-based cathode, defects, modification strategy

## Abstract

Li-rich manganese-based oxide (LRMO) cathode materials are considered to be one of the most promising candidates for next-generation lithium-ion batteries (LIBs) because of their high specific capacity (250 mAh g^−1^) and low cost. However, the inevitable irreversible structural transformation during cycling leads to large irreversible capacity loss, poor rate performance, energy decay, voltage decay, etc. Based on the recent research into LRMO for LIBs, this review highlights the research progress of LRMO in terms of crystal structure, charging/discharging mechanism investigations, and the prospects of the solution of current key problems. Meanwhile, this review summarizes the specific modification strategies and their merits and demerits, i.e., surface coating, elemental doping, micro/nano structural design, introduction of high entropy, etc. Further, the future development trend and business prospect of LRMO are presented and discussed, which may inspire researchers to create more opportunities and new ideas for the future development of LRMO for LIBs with high energy density and an extended lifespan.

## 1. Introduction

The twin problems of the energy crisis and environmental pollution have been the most serious challenges for the development of the global community in recent years. The main focus of research and development is on seeking a combination of theoretical research and experimentation to address these two major challenges, thereby realizing a new generation of more environmentally friendly energy technologies [1,2,3,4]. One focus is on the conversion and storage of clean energy, while lithium-ion battery (LIB) systems are one of the most anticipated energy storage devices [5,6,7]. LIBs have the advantages of low manufacturing cost, low weight, high energy density, no memory effect, less self-discharge, a durable charge/discharge cycle life, and high safety. Given such significant factors, LIBs have been widely used in computer, communication and consumer electronic products, and new-energy vehicles [8,9,10].

Currently, the cost of manufacturing cathode materials accounts for 40% of the total cost of an LIB. Typically, the energy density of an LIB is mainly influenced by the specific capacity and working voltage of the cathode material, as the cathode is the supplier of Li^+^. Currently known cathode materials mainly include lithium cobaltate (LiCoO_2_), lithium iron phosphate (LiFePO_4_), lithium manganate (LiMn_2_O_4_), and layered ternary materials (NCM) [11,12,13,14,15,16]. At present, the reversible specific capacity of commercial carbon anodes is higher than 350 mAh g^−1^, which is much higher than that of the above-mentioned cathode materials, while the theoretical specific capacity of emerging silicon-based cathode materials is as high as 4200 mAh g^−1^, which is about 10 times higher than that of graphite cathode [17,18,19,20]. Therefore, the development of high-specific-capacity cathode materials is an effective way to improve the energy density of LIBs [21,22].

Compared with the cathode materials mentioned above, layered Li-rich manganese-based oxide (LRMO) cathode materials with the chemical formula xLi_2_MnO_3_·(1−x)LiTMO_2_ (TM = Ni, Mn, Co, etc.) have attracted much attention, due to their excellent specific capacity (exceeding 250 mAh g^−1^) and considerable energy density (more than 1000 Wh kg^−1^), thus being considered as an ideal cathode material for future electric vehicle power batteries [23,24,25,26,27]. However, the defects of phase transition [28], O_2_/Li_2_O generation [29], interfacial side reactions, and transition metal (TM) dissolution [30,31] make the LRMO cathode face three basic challenges in practical applications: (i) low initial coulombic efficiency (ICE); (ii) rapid capacity and voltage decay; and (iii) poor multiplicative performance, all of which limit the practical commercialization of LRMO.

To tackle the aforementioned issues, numerous scholars have employed various techniques such as surface coating [32], elemental doping [33], and structural design [34], etc. in order to suppress the occurrence of lattice oxygen precipitation, migration of TM elements, and irreversible phase transition at the structural level, etc. These modifications have indeed brought about a certain enhancement in the performance of ICE, as well as an improvement in their cycling stabilities, while facile, cost-effective, and high-efficient strategies are urgently sought after, which drives the global researcher to explore further.

**Figure 1:**
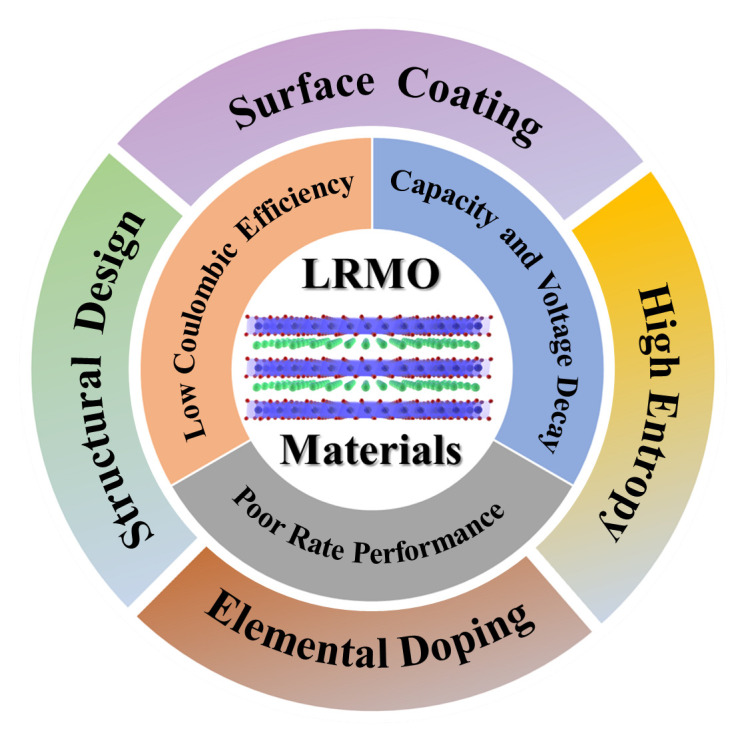
Outline schematic diagram of the problems with LRMO materials and corresponding effective solutions.

This review presents the most recent research progress in LRMO in terms of crystal structure, redox mechanism and electrochemical performance, and summarizes the inherent material issues and external performance-degradation challenges hindering its commercialization, as well as the current applied solution strategies (Figure 1). Here we provide perspectives and guidance for the researchers for the future investigation and development of LRMO, thereby achieving the overall improvement in electrochemical performance through effective modification strategies, which will ultimately promote the practical application of LRMO.

## 2. Material Structure and Reaction Mechanism of LRMO

**Figure 2:**
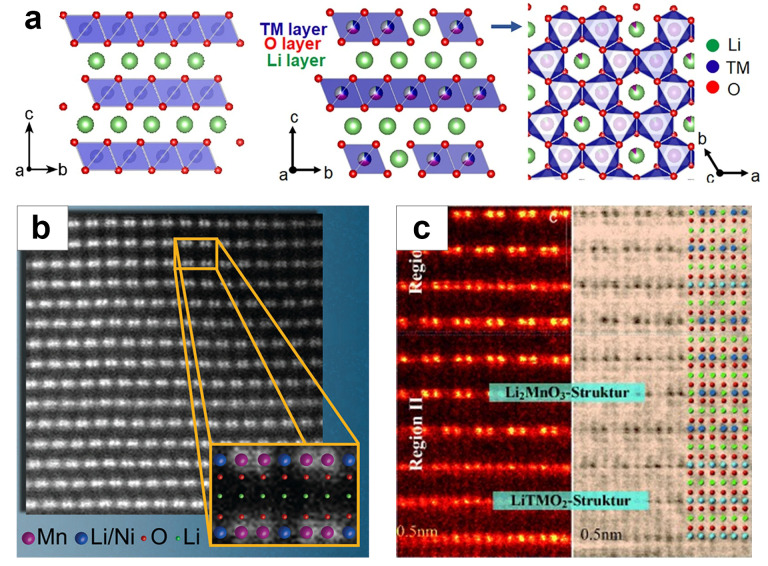
(**a**) Structure diagram of LiTMO_2_ and Li_2_MnO_3_ [35]; (**b**) aberration-corrected STEM image of a Li[Li_0.2_Ni_0.2_Mn_0.6_]O_2_ crystal [41]; (**c**) HAADF and ABF-SEM of LiTMO_2_ and Li_2_MnO_3_ [43].

The structure of electrode materials is intimately linked to their electrochemical properties and reaction mechanisms, underscoring the importance of understanding the intrinsic structure features of Li-rich compounds. LRMOs are derived from LiTMO_2_ (where TM represents Ni, Co, Mn, etc.). In LiTMO_2_, Li^+^ and TM ions alternate in occupying octahedral positions along the c-axis, forming a cubic close-packed array of oxygen (space group R-3m). When some of the TM ions are replaced by Li^+^, LiTMO_2_ transforms into LRMO. Interestingly, a locally ordered honeycomb structure (LiMn_6_) forms in the TM layer due to the reduced electrostatic repulsion between Mn^4+^ and Li^+^. This structure bears a resemblance to the structure of Li_2_MnO_3_ (space group C2/m) (refer to Figure 2a) [35]. Consequently, the academic community has yet to reach a consensus on the structure of LRMO. Up until now, two structural models have been proposed: (i) a single-phase solid solution model and (ii) a two-phase composite model.

The single-phase solid solution model Li[Ni_x_Li_(1−2x)/3_Mn_(2−x)/3_]O_2_ (0 < x < 1/2) was firstly proposed by Dahn et al. [36]. When x is a certain value, Li atoms are arranged in the TM layer and TM atoms are uniformly distributed in the remaining TM layer positions. By studying the X-ray diffraction (XRD) data of different fractions of LRMO, the researchers found that the Bragg peaks move smoothly with the fractions, which is consistent with Vegard’s law describing the solid solution phase. Thackeray et al. [37]. proposed a two-phase composite model based on previous studies, with the component symbols expressed as xLi_2_MnO_3_·(1−x) LiTMO_2_. The octahedral positions (space group R-3m) of the closely spaced oxygen arrays in LiTMO_2_ are occupied by alternating Li and TM layers, and the structure of Li_2_MnO_3_ (space group C2/m) is similar to that of LiTMO_2_, while the Li^+^ occupy one third of the TM positions. According to their test results, both of the interlayer distances between the (001) facet of the Li_2_MnO_3_ phase and the (003) facet of the LiTMO_2_ phase are close to 4.7 Å. However, it is difficult to distinguish the two phases by high-resolution transmission electron microscopy (HRTEM), due to the overlapping of the lattice fringes. When considering XRD characterizations, due to the structural compatibility of these two-layer phases, all the strong diffraction peaks in the XRD plots can be labelled as the R-3m space group, while the weak diffraction peaks in the 20–25° range can be labelled as the C2/m space group. In addition, under the circumstance of the low Li content and presence of Co, the characteristic diffraction peaks of Li_2_MnO_3_ are difficult to detect by XRD. Therefore, the employment of magic angle spinning–nuclear magnetic resonance (MAS-NMR) [38] spectroscopy and convergent electron beam diffraction (CEBD) is essential, so that the corresponding signals can be clearly collected [39]. However, it cannot be neglected that the XRD data merely provide information for the average crystal structure of the samples, so more direct evidence is sought to further elucidate the crystal structures [40].

Advanced characterization techniques have been the key methods for obtaining information about the crystal structure of LRMO. Many advanced monitoring and detection methods, such as time-of-flight neutron diffraction (TOF ND), high-resolution transmission electron microscopy (HRTEM), combined electron energy loss spectroscopy (EELS), aberration-corrected scanning transmission electron microscopy (STEM), and high-angle annular dark-field scanning transmission electron microscopy (HAADF-STEM), have been used to obtain the structural information. For the first time, combining diffraction scanning transmission electron microscopy (D-STEM), aberration-corrected scanning transmission electron microscopy (STEM), and STEM computer simulations, Jarvis et al. [41] demonstrated that forms a solid solution forms at the atomic level in Li[Li_0.2_Ni_0.2_Mn_0.6_]O_2_ (Figure 2b). According to their results, the crystal structure of the material belongs to the C2/m symmetry space group. Given neutron diffraction (ND) is more sensitive to light elements, especially Li and H, than XRD, Meng et al. [42] performed Rietveld refined TOF ND maps, which not only precisely calculated the detailed unit cell parameters of the LRMO cathode material, but also accurately obtained the number of oxygen vacancies in the particles. In addition, the ND results indicate that the layered LRMO is actually a solid solution of the LiTMO_2_ phase and Li_2_MnO_3_ phase. Yu et al. [43] employed atomic resolution STEM to provide convincing evidence for the coexistence of a rhombic LiMO_2_ structure and a monoclinic Li_2_MnO_3_ structure in the Li_1.2_Mn_0.567_Ni_0.166_Co_0.067_O_2_ material. More precisely, the microregions containing these two phases are separated by the (001)rh/(103)mon plane along the [001]rh/[103]mon direction, proving the local structural variations of the LRMO cathode material, which is not simply a single solid solution (Figure 2c).

In addition to the similarity and complexity of the structures of the two phases, LiTMO_2_ and Li_2_MnO_3_, the factors that make it difficult to determine the crystal structure of LRMO are also related to the different material compositions and synthesis conditions, such as the lithium content, calcination conditions, and cooling rate [8,44]. Meanwhile, the combination of advanced characterization techniques with theoretical simulations has the potential to develop a new system to compensate for the shortcomings of different methods, which will be an important breakthrough in designing the crystal structure of LRMO. The study of the structure of LRMO valence bond structure, energy band structure, and other structures has greatly facilitated the exploration of the corresponding electrochemical reaction mechanism, but the controversy about the compositional structure of LRMO may still continue.

**Figure 3:**
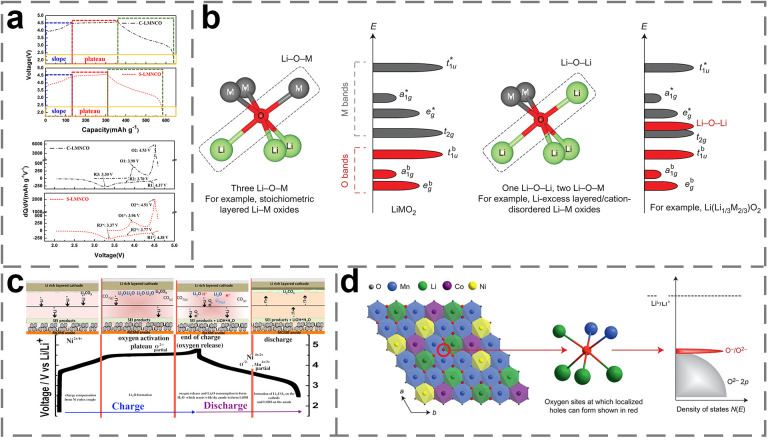
(**a**) The initial charge–discharge curve of LRMO [44]; (**b**) surface reaction mechanism of oxygen-activated molecules during charge and discharge [52]; (**c**) structural and chemical origin of the preferred oxygen oxidation along the Li-O-Li configuration [29]; (**d**) schematic of local structure and energy versus DOS which shows position of O^−^/O^2−^ redox couple of LRMO with 3d TM_3_ [21].

Due to the unique crystal structure of LRMO, the redox mechanism is more complex compared to other cathode materials [45]. Figure 3a shows a typical LRMO first-turn charge/discharge curve, from which it can be seen that the first charging curve can be divided into two parts, below 4.5 V and above 4.5 V. The first charging curve can be divided into two parts above 4.5 V. An obvious inclined potential curve can be observed from the open circuit voltage to 4.5 V. The charging and discharging characteristics of this part of the curve are similar to those of the ternary cathode. In this reaction, lithium ions are detached from the electrochemically active LiTMO_2_ component with the occurrence of Ni^2+^/Ni^4+^ and Co^3+^/Co^4+^ oxidation reactions, corresponding to the first oxidation peak in the CV curve. At the same time, the lithium ions occupying the octahedral positions of the Mn layer in the Li_2_MnO_3_ phase diffuse to the tetrahedral positions of the Li layer in the LiTMO_2_ phase, which contributes to the structural stability. A long voltage plateau occurs at 4.5 V. This is due to the activation of the electrochemically inert Li_2_MnO_3_ structure, which provides a higher capacitance due to Li delocalization from its lattice and oxidation of the lattice oxygen, but there is no further oxidation of Mn^4+^ in the structure and no change in the valence state of the other TM elements. Initially, researchers universally attribute the structure degradation of LRMO to the formation of Li_2_O during oxidation and the irreversible conversion from activated inert Li_2_MnO_3_ to active MnO_2_ (Figure 3b). During the subsequent discharge process, Li^+^ can reinsert into the MnO_2_ structure until the LiMnO_2_ forms. Obviously, two Li^+^ escape from each Li_2_MnO_3_ unit during charging, while only one Li ion can be reversibly embedded in the subsequent discharging process, which implies that the first-loop irreversible capacity is unavoidable. Therefore, in addition to serving as an additional Li source and structural stabilization pillar, the Li_2_MnO_3_ phase in the LRMO determines the amount of electrochemically active metal in the electrode after the activation above 4.5 V. The activation process seldom occurs completely during the first charge cycle [29,46,47,48]. Sung et al. [49] confirmed the existence of a continuous activation process of Li_2_MnO_3_ remaining even after the first cycle, and their results suggested that it is responsible for the capacity increase in the first few cycles and the voltage decay during the cycle, as well. Liu et al. [50] investigated the charge/discharge voltage and reaction mechanism of LRMO based on first-principles calculations and confirmed that the asymmetric oxidation behavior exits at the Li_2_MnO_3_/LiTMO_2_ interface, which facilitates higher voltages for LRMO compared to the pure layered oxide cathode. Che et al. [51] investigated the content ratio of Li_2_MnO_3_/LiTMO_2_, and their results showed that a reasonable ratio is required to achieve a balance between high capacity and stable cycling.

The reason for the extremely high capacity of LRMO cathode materials is in part due to the involvement of anions in the redox process. Initially, it was widely believed that the anion redox phenomenon was induced by the change and rearrangement of the electronic orbital structure of the TM with oxidation, leading to the formation of perovskite-like oxides in the transition metal layer. Later, researchers tended to believe the theory of anionic redox mechanism based on the positions of the electron oxygen orbitals. Using advanced computational and analytical techniques, the researchers proposed that anionic redox occurs under the following conditions: (i) when non-perovskite-like bonding orbitals of O (unhybridized O orbitals) are present between the hybridized O-bonding state and the anti-bonded TM state in the TMO structure, due to the formation of Li-O-Li bonds and (ii) when the O–M interactions are relatively ionized, this leads to localized O 2p orbitals in the transition metal layer. In this case, the anionic redox produces localized electron holes on the O atoms. According to the research by Ceder et al. [52], it is shown that in the Li-O-Li configuration of the LRMO system, O 2p does not hybridize with Li due to the energy difference, and the orbitals of O produce unhybridized orbital-like states (Figure 3c). The reversible capacity is increased, since the new unhybridized energy band is above the bottom of the TM energy band. This is because electrons can transfer not only from the TM band but also from the unbonded states. This theory can explain the cationic and anionic redox properties of Li-rich layered oxides, where anionic redox can be separated from the cationic redox. However, anionic redox has been reported to occur even in structures without the Li-O-Li configuration. Given that additional anionic redox mechanisms demand further explanation of this phenomenon, Bruce et al. [23] proposed an anion redox mechanism based on a localized oxygen vacancy model. In their study, it was shown that in LRMOs consisting of only 3d TMs, the O-(Mn^4+^/Li^+^) interactions are less covalent and have localized O 2p orbitals that can provide electrons. As shown in Figure 3d, due to the ionic O–Mn^4+^ interactions, the electron holes generated by the O 2p electron transfer are located on the O atoms. Given that the oxygen redox reaction occurs with the absence of excess alkali metal ions, it is concluded that the anions in the O–TM interactions can undergo redox in a reduced covalent manner, even under the circumstances of absence of the Li-O-Li configuration in LRMO.

Despite the fact that anionic redox can provide extra capacity to achieve high energy density of LRMOs, its irreversibility leads to inevitable cation migration and collapse of the layer structure. Research on anionic redox is still in its infancy and needs to be further explored to obtain more stable high-capacity cathode materials for LIBs with an extended lifespan.

## 3. Defects in the LRMO

Considering the relatively high charging potential, the anionic participation in redox reactions, as well as the direct contact of the material with the electrolyte, LRMO will inevitably undergo side reactions such as lattice oxygen loss, irreversible phase transition (layered→spinel), and dissolution of TM ions, resulting in low ICE, severe capacity and voltage decay, and poor multiplicative performance of LRMO [53].

### 3.1. Low Initial Coulombic Efficiency

To date, most LRMO cathodes have a typical ICE of less than 85% due to their unique charging and discharging mechanisms, which is far below the practical application targets (>90%). As mentioned above, when the charging potential of the LRMO is higher than 4.5 V, a voltage plateau occurs, representing the redox of O^2−/−^ anions and the detachment of Li from the TM layer [54]. The genesis of O-O dimers is the formation of coplanar 2p unhybridized orbitals of two neighboring Os, and this particular configuration triggers an additional high-energy energy band structure and anionic charge compensation behavior during the downward shift of the Fermi energy level, leading to the decrease in the ICE [55]. At the same time, the activation of Li_2_MnO_3_ begins, which is a process accompanied by the irreversible loss of oxygen and the appearance of the active species MnO_2_ and the production of the inactive species Li_2_O. Due to the combination of the exfoliated Li^+^ with O^2−^ in the lattice forming the inactive component Li_2_O, Li^+^ vacancies are created and the transition metal ions will migrate and occupy the Li vacancies, resulting in the irreversible reverse of Li^+^ to the Li vacancies, thereby leading to the decrease in the initial capacity [52,56].

### 3.2. Excessive Capacity and Voltage Drop

**Figure 4:**
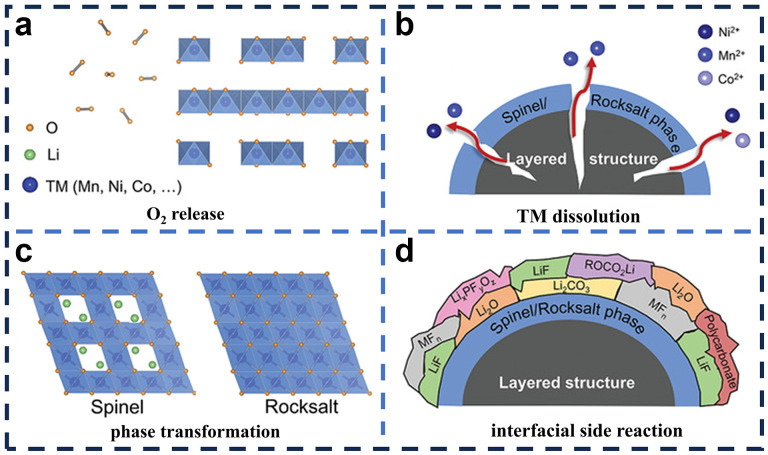
Schematic diagram of structural degradation on the surface of LRMO [53]. (**a**) Irreversible release of lattice oxygen; (**b**) dissolution of transition metals; (**c**) irreversible changes in phase structure; (**d**) interfacial side reaction.

At the end of the oxidation process, due to the difficulty in controlling the precipitation of lattice oxygen, the O^2−/−^ is partly oxidized to oxygen, some of which is trapped in the bulk phase while some is released through the surface of the LRMO (Figure 4a). During the subsequent discharge, the released O_2_ can only partially be reduced to O^2−^, resulting in low ICE and the hysteresis phenomenon of the voltage. Prolonged cycling also leads to penetration of oxygen defects into the crystal and collapse of the bulk structure, which promotes further loss of O_2_ from the LRMO, formation of microstructural defects, and acceleration of the voltage decay [57,58].

It is generally believed that the release of lattice oxygen during charging occurs in the Li_2_MnO_3_ phase, leading to the formation of MnO_2_ or Li_x_MnO_2_. This process is acknowledged to be irreversible during the subsequent discharge. At the same time, it is believed that the excessive shedding of Li further leads to the migration of TM ions and induces the structural transition from the layered to the spinel phase. As shown in Figure 4b, due to the different crystal structures of the layered and spinel phases, the arrangement of Li and the migration of TM ions are involved in the phase transition process. Specifically, when Li^+^ migrate during the charging process, TM ions spontaneously and continuously migrate to the Li sites in the Li and TM layers, which makes it difficult for Li^+^ to be embedded during discharge, and then causes irreversible phase transitions from laminar to spinel, resulting in the degradation of voltage and battery capacity [59,60,61]. In addition, these phase transitions initially occurred on the surface of LRMO and then gradually transferred to the grain interior in the subsequent cycles, resulting in the destruction of the internal lamellar structure of the grains, the concentration of stresses at grain boundaries, the formation of cracks, and even grain crushing. Consequently, the destruction of the structure is widely believed to be the main cause of capacity and stress degradation.

### 3.3. Poor Rate Performance

The chemical valence of TM gradually decreases due to O_2_ release and irreversible phase transitions. Reduced TM ions are typically generated in the LRMO, which is aged after cycling, and then undergo a dissolution–migration–deposition process (Figure 4c) [62]. In detail, TM ions are first dissolved from the cathode electrode, then migrated through the electrolyte, and finally deposited on the anode electrode. In addition, the reduction–deposition of TM ions on the anode electrode increases the thickness of the solid electrolyte interfacial layer, which induces a higher battery impedance, thus hindering the transport of Li^+^ [63].

At the high charging voltage of 4.8 V, the LRMO interface, which is in direct contact with the electrolyte, is extremely unstable and spontaneously undergoes side reactions. During the repeated charging/discharging process, various types of compounds such as LiF and Li_2_CO_3_ form on the surface of the positive electrode, forming a thick electrolyte interface layer (CEI) (Figure 4d). The overgrown CEI prevents the diffusion of Li^+^ and thus reduces the electrochemical performance [64].

## 4. Modification Strategies

The problems of lattice oxygen loss, irreversible phase transition, TM ion dissolution and interfacial side reactions have severely limited the commercial use of LRMO. In the past 20 years, a great deal of research has been devoted to solving these problems; e.g., structural design, ion doping, surface coating, and other modifications have been proposed to improve the electrochemical performance of LRMO [32,53].

### 4.1. Surface Coating

Surface coatings can adequately protect the electrode materials from electrolyte erosion, and impede lattice oxygen release and phase transitions, so surface coatings are one of the most widely studied methods for LRMO modification [65]. Coating materials should meet the following requirements: (i) high stability of physical and chemical properties; (ii) resistance against electrolyte corrosion; and (iii) high ionic conductivity. The major LRMO surface coating materials reported to date include oxides [66], fluorides [67], phosphates [68], Li^+^ conductors [69], and conductive polymers [70].

**Figure 5:**
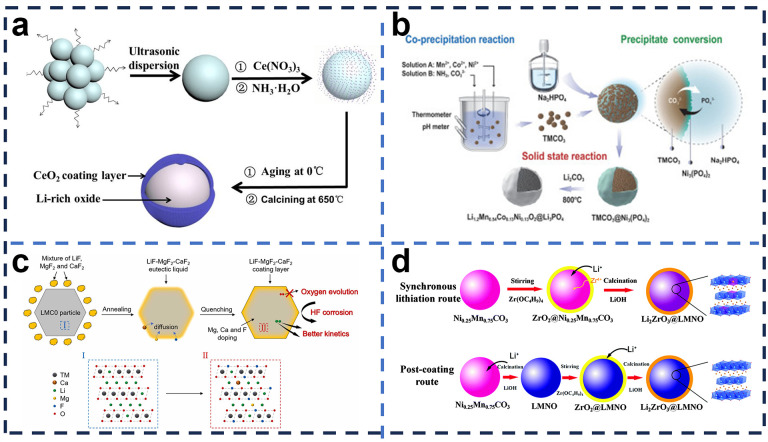
(**a**) Schematic of CeO_2_ coating modification [76]; (**b**) schematic of LiF-MgF_2_-CaF_2_ coating modification [83]; (**c**) schematic of Li_3_PO_4_ coating modification [85]; (**d**) schematic of Li_2_ZrO_3_ coating modification [90].

Oxide has a stable structure and is a typical coating material that prevents side reactions between the active material and the electrolyte, and thereby inhibits oxygen loss effectively. It can also react with HF to reduce the content level of HF in the electrolyte and reduce the dissolution of transition metals, thus improving the stability of the layered structure of the cathode material. Currently, well-known metal oxides for capping include Al_2_O_3_ [71], ZnO [72], MnO_2_ [73], etc. For instance, ZrO_2_-coated LRMO materials were prepared by the sol–gel assisted ball-milling method by NISAR et al. [74]. The ZrO_2_-coated LRMO materials exhibited higher capacity retention, better multiplicity performance, and more excellent thermal stability. Their improved electrochemical performance can be attributed to the stabilization of their surface structure. Pan et al. [75] used Nb_2_O_5_ to coat LRMO, and the coated material showed 98% capacity retention after 200 cycles at 1C (1C = 250 mAh g^−1^). In addition, it exhibited excellent multiplicative performance, delivering 189 and 152 mAh g^−1^ at high current densities of 2C and 5C, respectively. This is because Nb_2_O_5_ not only acts as a fast ionic conductor to accelerate the diffusion of Li^+^ at the cathode/electrolyte interface, but also acts as an inert protective layer to reduce the direct contact between the cathode and the electrolyte, thus suppressing the phase transition and voltage decay. Liu et al. [76]. coated a Co-free Li-rich oxide with a layer of CeO_2_ using a low-temperature aging process (Figure 5a), obtaining a composite material with improved cycling stability and enhanced rate capability as a cathode for LIBs.

Metal oxide coatings can convert into metal fluorides by HF corrosion during cycling, thus further protecting the cathode material [77]. Therefore, fluorides (AlF_3_ [78], MgF_2_ [79], LiF [80], etc.) can be used directly for cladding modification. On one hand, the commonly used electrolyte for LIB is LiPF_6_, and F^−^ can effectively inhibit the occurrence of interfacial side reactions [81]. On the other hand, the addition of F^-^ reduces the charge transfer resistance and increases the conductivity, which improves the multiplicity and cycling performance of the cathode materials [82]. Zhang et al. [78] prepared AlF_3_ by high-temperature solid-phase reaction. In their preparation, AlF_3_ was uniformly coated on a Li-rich Mn-based cathode. Charge–discharge cycling tests were conducted in the voltage range of 2.0–4.8 V. The specific capacity of the AlF_3_-coated Li-rich manganese-based cathode material was significantly increased to 283.3 mAh g^−1^, while the capacity retention rate was still 84.4% after 200 charge–discharge cycles. During the charge/discharge process, AlF_3_ can maintain the stability of the main cathode material and inhibit the further transition, thus ensuring the high specific capacity and cycling stability of the material. Using a simple eutectic molten salt method, Zhao et al. [83] synchronized passivated fluoride coating and functional doping in cobalt-free lithium iron-rich cathode Li_1.2_Ni_0.13_Fe_0.13_Mn_0.54_O_2_ by controlling the melting and solidification processes of LiF-MgF_2_-CaF_2_ ternary salt, as shown in Figure 5b. The outer fluoride layer effectively inhibited the release of oxygen and prevented the dissolution of TM ions, while the internal doping elements improved the diffusion kinetics of Li^+^ and further stabilized the crystal structure, resulting in a significant improvement in the electrochemical performance of the modified cathode material.

The chemical bond between the polyanion PO_4_^3−^ and the metal ions in the phosphate is strongly covalent [83,84], which can reduce the contact between the LRMO and the electrolyte, thus improving the structural and thermal stability of the material. Meanwhile, the solid electrolyte layer formed by Li^+^ and phosphate facilitates ion diffusion, which effectively improves the Li^+^ transport efficiency at the electrode/electrolyte interface and enhances the electrochemical performance of the material. Liu et al.[85] achieved an in situ lithium phosphate coating modification and modified Li_1.2_Mn_0.54_Co_0.13_Ni_0.13_O_2_ for LRMO LIB cathode materials by the “carbonate co-precipitation–precipitation conversion–solid-phase reaction” method (Figure 5c). The capacity of the assembled half-cell reached 191.1 mAh g^−1^ after 175 cycles at a current density of 125 mA g^−1^, with a capacity retention rate of 81.8% and an average voltage decay of only 1.09 mV per cycle. The lithium phosphate coating layer mitigated the side reaction between the material surface and the electrolyte, thereby suppressing the irreversible phase transition and transition-metal dissolution. At the same time, lithium phosphate acted as a lithium conductor to promote the transport of Li^+^. Song et al. [86] presented an in situ hybridized phosphate coating that combines the advantages of excellent ionic conductivity (Li_3_PO_4_) and structural stability (LiTMPO_4_, TM = Ni, Mn) through ion exchange and thermal treatment. The ICE of the coated sample was 92.79% at 0.1C and the capacity retention after 100 cycles was 85.33%. This was attributed to the synergistic effect of Li_3_PO_4_ and LiTMPO_4_, which accelerated Li^+^ transport while reducing surface side reactions and the strong P–O bond, which inhibited irreversible oxygen release and stabilized the surface structure.

Li-ion conductor materials (Li_2_TiO_3_ [87], LiAlO_2_ [88], Li_2_MnO_3_ [89] etc.) have fast ion-diffusion paths and high electronic conductivity. In addition to protecting the electrodes from F-corrosion, this proper strategy can avoid unwanted side reactions at the electrode interfaces and stabilize the lamellar structure, thereby improving the rate performance, cycling performance, and thermal stability of the cathode materials. Zhang et al. [90] reported a new Syn-Li_2_ZrO_3_@LRMO material consisting of Li_2_ZrO_3_ coated with Li_1.2_Mn_0.6_Ni_0.2_O_2_ (LRMO). The synthesis process is shown in Figure 5d. The material combines the advantages of Li_2_ZrO_3_ coating and Zr^4+^ doping, and has higher multiplicity performance and cycling stability than the @LRMO material, which is first doped and then coated. This is because the coating reduces the surface side reactions and inhibits the dissolution of TM ions. The Zr^4+^ doping increases the crystal spacing and reduces the Li/Ni mixing, which is conducive to the diffusion of Li^+^. Meanwhile, the synergistic effect of Li_2_ZrO_3_ coating and Zr^4+^ doping further enhances the stability of the layered structure and reduces the voltage decay during cycling.

**Figure 6:**
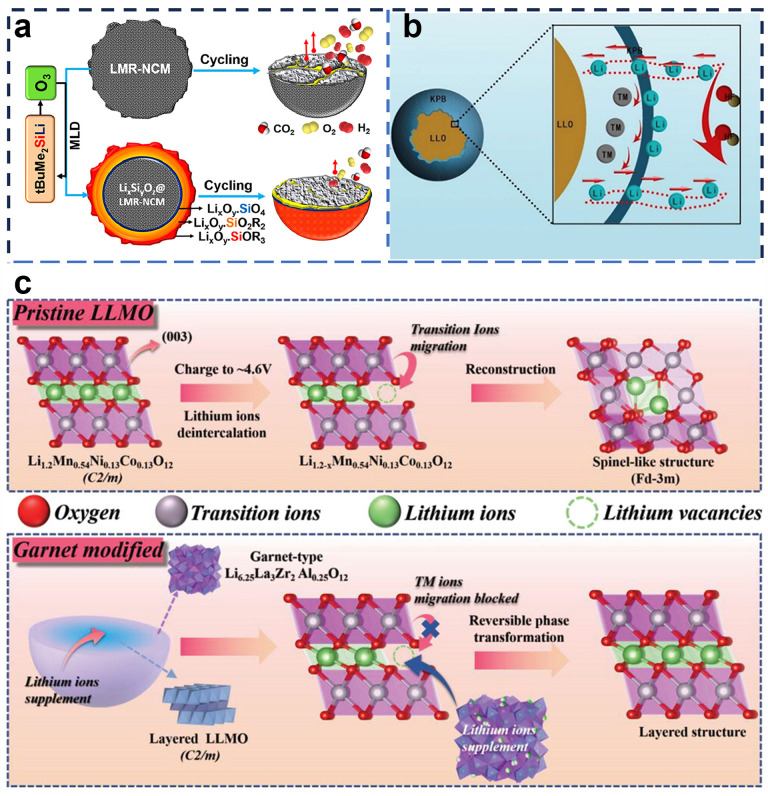
(**a**) Schematic of Li_x_Si_y_O_z_ coating modification [91]; (**b**) schematic of Prussian blue coating modification [92]; (**c**) schematic of LLZAO coating modification [93].

There are many new coating materials in addition to the main categories mentioned above. For instance, Rosy et al. [91]. introduced t-butyldimethylsilyl lithium as a single-source precursor for the deposition of Li_x_Si_y_O_z_ and an artificial cathode/electrolyte interface (ACEI) as a coating layer on the surface of the LRMO, which is schematically shown in Figure 6a. The energy density of the coated material was increased from 457 to 644 Wh kg^−1^, while the graphite/LMR-NCM full cell showed an increase in capacity of about 35% and a capacity retention of >80% over 200 cycles. Xu et al. [92] used the Li-ion receptor Prussian blue (KPB), which has good ionic conductivity, as a novel LRMO coating material. The reaction mechanism of the KPB coating is shown in Figure 6b. The KPB coating not only forms a protective layer on the surface of the LRMO to prevent electrolyte corrosion, but also acts as the main body of Li^+^ transport and accommodation, which enhances the ionic conductivity of the LRMO cathode and the ICE. Wei et al. [93] used Li_6.25_La_3_Zr_2_Al_0.25_O_12_ (LLZAO), a Li-ion conductor with excellent performance, as a coating on LRMO. The capacity retention of the modified sample was as high as 95.7% after 300 cycles at 1.0C, which was attributed to the excess Li^+^ in LLZAO mitigating the phase transition of spinel by inserting the lithium layer, thereby stabilizing the anode structure (Figure 6c). Ye et al. [94] constructed an integrated and stable carbon rock salt/spinel composite heterostructure layer (C@spinel/MO) by in situ self-reconfiguration, and due to the synergistic effect of the surface spinel phase and the Mo-type rock-salt phase, the integrated electrochemical performance of the lithium-rich manganese-based cathode material was significantly improved, and the surface stability of the material was greatly enhanced.

### 4.2. Elemental Doping

Elemental doping is considered one of the simplest modification methods. Ion doping inhibits cation mixing, forms a surface protective layer against electrolyte erosion, and acts as a carrier ion to widen Li^+^ diffusion channels. The purpose of doping with different elements is to improve the electrochemical properties by strengthening the crystal structure. According to the different types and positions of ion doping, it can be divided into cation doping, anion doping, and co-doping.

**Figure 7:**
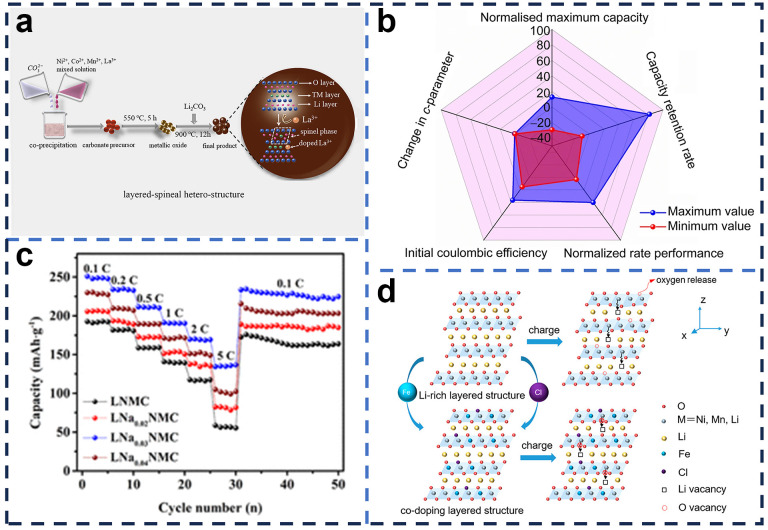
(**a**) Schematic diagram of La doping and the corresponding crystal structure [101]; (**b**) rate performance for LNMC and LNaxNMC [102]; (**c**) spider plot demonstrating spread of results achieved by bulk F-doping on important LRC performance metrics [109]; (**d**) schematic diagram of Fe and Cl co-doped LRMO [111].

Cation doping can be further classified into TM-layer doping substitutions (Ti^2+^, Zn^2+^, Al^3+^, Nb^5+^, La^3+^, etc.), Refs. [95,96,97,98,99] and Li-layer doping substitutions (Na^+^, K^+^, etc.) [100,101], based on the doping sites. Wang et al. [99] synthesized La-doped Li[Li_0.2_Mn_0.54_Ni_0.13_Co_0.13_]O_2_ cathode materials with excellent electrochemical properties (Figure 7a). The results showed that the addition of a suitable amount of La^3+^ and the corresponding layered spinel heterostructure phase to the Li-rich cathode material can significantly improve the cycling and multiplicity performance. This is due to the fact that the large radius of La^3+^ can promote the migration of Li^+^ through the extended layered structure. In addition, as an electrochemically inert element, La^3+^ does not participate in the electrochemical reaction and can further stabilize the structure of the material. Meanwhile, the induced spinel phase provides a three-dimensional diffusion channel that facilitates the migration of Li^+^, thus improving the multiplicity performance of the material. Zhang et al. [100] prepared Na-doped Li_1.23−x_Na_x_[Ni_0.2464_Mn_0.462_Co_0.0616_]O_2_ cathode materials by solid-phase reaction. The doping of Na^+^ can effectively mitigate the decrease in discharge capacity and operating voltage. The doping of Na^+^ in the layered structure increases the lattice-layer spacing, which contributes to the favorable diffusion of Li^+^ and slows down the precipitation of lattice oxygen. Meanwhile, Na^+^ doping further activated the Mn^4+^ in Li_2_MnO_3_ and provided reversible capacity in the subsequent cycles. Furthermore, the doped material has intrinsically superior rate properties itself, as shown in Figure 7b. Fan et al. [102] verified by synchrotron characterization the fact that Cr doping prevents Mn migration and reduces the original stable delithiated structure, while tetrahedral Cr spontaneously migrates back to the octahedral sites after delithiation, driven by energy interest. Therefore, the structural stability of Cr-doped Li_1.2_Ni_0.2_Mn_0.6_O_2_ at high delithiation is significantly enhanced, resulting in longer cycle life. Cong et al. [103] used a Ni/Mg double concentration-gradient-repair doping strategy, where the Ni/Mg content gradually decreases from the surface to the center of the material and the content of Mn gradually increases. The high Ni on the surface increased the ratio of cationic redox, increased the working voltage, and accelerated the reaction kinetics. In addition, Mg doping on the material surface inhibits the migration of transition metals, thus stabilizing the layered structure.

Anion doping can be simply divided into low-valent anion doping (F^−^, Cl^−^, S^2−^, etc.) [104,105,106] and polyanion doping (SO_4_^2−^, PO_4_^3−^) [107,108]. Seaby et al. [109] found that F^-^doping can significantly improve the four key properties of LRMOs, such as activation, capacity, cycling stability, and multiplicity performance, as shown in Figure 7c. F^-^doping significantly improves multiplicity performance and cycling stability, which may be due to the charge compensation and greater electronegativity. In addition, F^-^doping could increase the capacity by improving the reversibility of the anion redox. Zhang et al. [110] found that PO_4_^3−^ doping could stabilize the laminar structure of the material during long cycling and improve the cycling stability of the material due to the higher binding energy of PO_4_^3−^ towards the transition-metal cation.

Compared to separate cation or anion doping, anion–anion synergistic co-doping is more advantageous and is now the mainstream direction for material doping modification. Nie et al. [111] used Fe and Cl co-doping and cobalt-free LRMO (Figure 7d), and the results showed that Fe and Cl co-doping can promote the diffusion of Li^+^ and improve the rate performance of the materials. In addition, it was shown by first-principle calculations that Fe and Cl co-doping can improve the structural stability. The results showed that Fe and Cl co-dopants reduced the irreversible release of lattice oxygen, mitigated the voltage decay, and improved the ICE of the first cycle. Liu et al. [112] employed a Na and F co-doping method to improve the properties of Li_1.2_Ni_0.2_Mn_0.6_O_2_, a cathode material for LIBs. In this effective synthetic route, appropriate amounts of Na and F were doped at the Li and O sites, respectively, and the Na-doped samples exhibited excellent cycling stability due to the improved structural stability, while the F-doped samples showed higher capacity and better multiplicity performance, mainly due to the enhanced electronic and ionic conductivity.

### 4.3. Structural Designs

**Figure 8:**
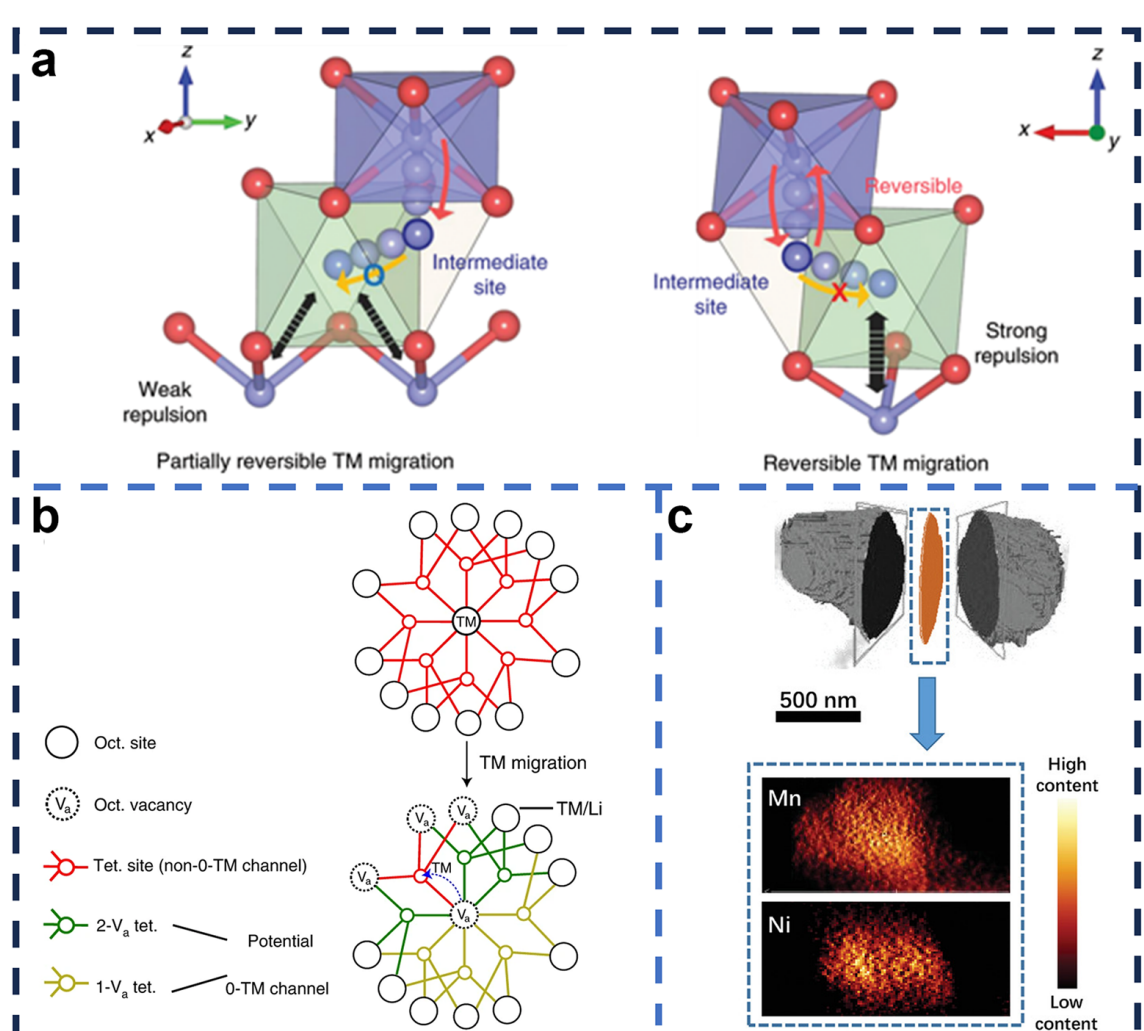
(**a**) Schematic illustrations of crystal structures of O3-type and O2-type lithium layered oxides [117]; (**b**) illustration of Li-pathway availability in a rock-salt-type structure before and after the TM migration [118]; (**c**) a “single-crystal” particle from the synchrotron soft X-ray imaging, with a selected equatorial slice from the center of the particle [119].

The crystal structure has a major influence on the electrochemical properties of LRMO. Li et al. [113] proposed a multifunctional full-interface integration project to introduce multifunctional modified layers (surface S- and N-doped carbon layers, near-surface gradient oxygen vacancies, and the resulting atomic rearrangements) at the interface between the secondary particles and the internal primary particles of LRMO. Oxygen vacancies and intra-structural Li/Mn disorder can inhibit oxygen release, while the induction of lattice-matched rock-salt phases can improve the stability of the interfacial structure. Li et al. [114] controllably introduced cationic disordered nanostructured domains into layered lithium-rich oxides by co-doping with d^0^-Tm and basic ions. Combined with advanced characterization by electrochemistry and synchrotron radiation, the introduction of compatible cationic disordered structural domains not only prevents the collapse of the oxygen skeleton along the c-axis of the layered lithium-rich cathode at high operating voltage, but also promotes the activity of Mn and anion, as well as the embedding kinetics of Li^+^, resulting in a significant improvement in the multiplicative capacity and a slowing down of the capacity and voltage decay. O2-type Li_2/3-1/3_Ni_0.25_Mn_0.75_O_2_ (LNMO-HT) was prepared by Li^+^/Na^+^ ion-exchange reaction and low-temperature heat treatment at 300 °C by Tang et al. [115]. High-resolution electron microscopy (HRTEM) images combined with geometric phase analysis (GPA) showed that LNMO-HT has the effect of suppressing lattice distortion and reducing microstrain, which is favorable for charge transfer and contributes to mitigating voltage decay. Jing et al. [116] designed and synthesized “Li-rich Ni-rich” with a core-shell structure consisting of a less active “Li-rich Mn-rich” shell and a high-capacity “Li-rich Ni-rich” core. As Li ions gradually enter the core-shell precursor during the high-temperature lithiation reaction, the elements sequentially interdiffuse at the interface between the Mn-rich shell and the Ni-rich core. This thermally driven interdiffusion of atoms results in a “Li-rich-Mn-rich” shell layer of controllable thickness, which ensures a special structural reversibility of the layered “Li-Ni-rich” cores during long-term cycling. Donggun Eum et al. [117] prepared an O2-type structure LRMO by changing the order of oxygen stacking in the layered structure (Figure 8a), which limited the migration of transition metals within the Li layer, inhibited the formation of O-O dimers, and facilitated the high-potential anion reduction, thus reducing the voltage hysteresis. Huang et al. [118] found that when the topological reaction of the lithium-rich cationic disordered rock-salt anode is replaced by a non-topological reaction, the fast non-topological lithiation reaction enables reversibility of transition-metal migration from octahedral to tetrahedral, resulting in a significant improvement in multiplicity performance. Schematic diagrams of lithium-ion channels in rock-salt-type formations before and after TM migration are shown in Figure 7b. Wu et al. [119] designed and prepared a concentration-gradient “single crystal” LRMO using a combination of co-precipitation and molten-salt sintering methods, with a gradual decrease in Mn content from the center to the surface and a gradual increase in Ni content (Figure 8c). On this condition, a capacity-retention rate of 97.6% and an energy density of 95.8% at 0.1C for 100 cycles can be achieved. The capacity-retention rate is 97.6% and the energy density is 95.8% after 100 cycles at 0.1C, and there is no crack emerging after long-term cycling. As a consequence, the phase transition is suppressed, which improves the thermal stability significantly.

### 4.4. Introduction of High Entropy

**Figure 9:**
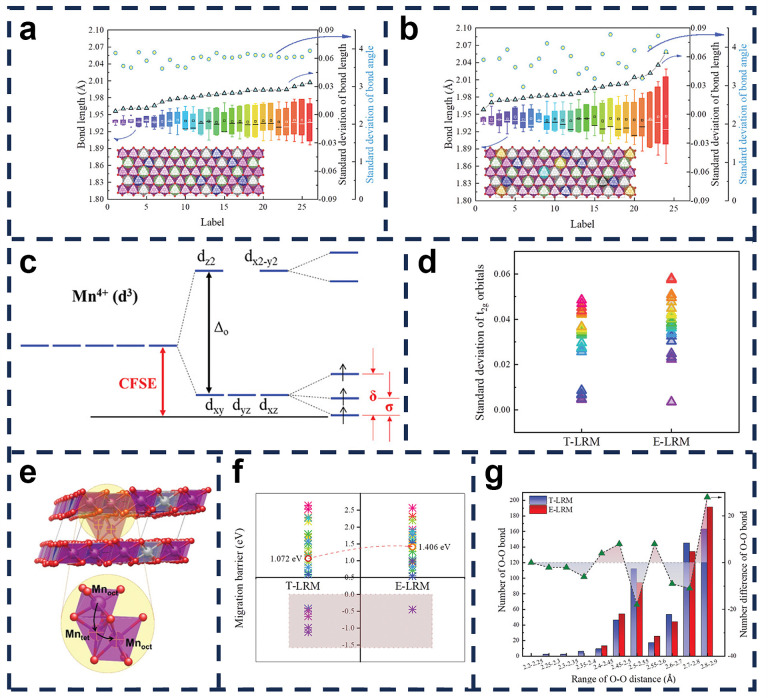
The distribution of Mn-O bond length and O-Mn-O (angle ≈ 90°) deviations in each MnO_6_ octahedron of (**a**) T-LRM and (**b**) E-LRM. The inserts are the corresponding optimized structure. (**c**) The schematic illustration of Mn^4+^ 3d orbital splitting in an octahedral crystal field. (**d**) The standard deviations of the energy center of three t2g orbitals for each Mn ion in T-LRM and ELRM. (**e**) The structural diagram of Mn-ion migration. (**f**) Energy barrier of each Mn-ion migration. (**g**) The distribution of O-O distance within octahedrons at fully delithiated states [109].

The introduction of high-entropy materials into LIB electrodes can optimize the various properties of LRMOs, which can help to improve structural stability, capacity retention, low- and high-temperature performance, ionic conductivity, etc. [120,121,122,123]. Various transition metals are mixed together to provide a stable crystal structure and uniform distribution of metal elements, which facilitates the generation of vacancies, thereby increasing ionic conductivity and improving the electrochemical performance of the battery. Song et al. [124] employed a high-entropy (when the configurational entropy is greater than 1.5 R, it can be referred to as a high-entropy material) doping strategy to overcome the stability–capacity trade-off problem of lithium-rich cathode materials. The designed entropy-stabilization strategy enhanced lithium-rich cathode material (E-LRM) has the chemical formula Li_1.0_[Li_0.15_Mn_0.50_Ni_0.15_Co_0.10_Fe_0.025_Cu_0.025_Al_0.025_Mg_0.025_]O_2_ and its octahedral TM sites are shared by eight elements. The introduction of multiple elements increases the local structural diversity and distortion energy of Mn^4+^, which is confirmed by the results of density functional-theory calculations (Figure 9), resulting in better local structural adaptation and improved structural stability. Zhang et al. [125] developed a zero strain and zero-cobalt layered anode, LiNi_0.8_Mn_0.13_Ti_0.02_Mg_0.02_Nb_0.01_Mo_0.02_O_2_ (HE-LNMO), using a novel doping strategy and a typical co-precipitation method. Compared to most state-of-the-art high-Ni and low-Co anodes, HE-LNMO exhibits unprecedented zero volume change during Li^+^ embedding and de-embedding, while achieving zero strain and high capacity, spontaneously. Due to its highly stable structure, HE-LNMO exhibits significantly higher capacity retention (85% at 1000 half-cell cycles) compared to commercial LiNi_0.8_Mn_0.1_Co_0.1_O_2_ (NMC-811).

## 5. Discussion

In order to meet the performance requirements of electric vehicles for next-generation battery systems, it is crucial to develop advanced batteries with high energy density, low cost, long cycle life, environmental friendliness, and high safety standards. LRMO material is one of the potential cathode material candidates for next-generation power LIBs due to its high discharge capacity, high energy density, and environmental friendliness. In this review, the reasons for the low ICE, excessive voltage/capacity decay, and poor multiplicity performance of LRMO are explained by the crystal structure of the material, as well as the reaction mechanism. At the same time, various modification methods from recent years are described in detail. The effectiveness of the various approaches is summarized in Table 1. With the improvement in proper modification methods and the advancement of thorough characterization methods, the electrochemical performance of LRMO materials has been continuously improved, and the understanding of their structural-evolution mechanism and charge-compensation mechanism has been further deepened. However, LRMO materials are still not comparable to typical commercial cathode materials in terms of voltage retention, cycling stability, and multiplicity performance, which demands that researchers make further progress.

Regarding the future development direction of LRMO materials, the following findings are presented and summarized in this review: (i) the dynamic coupling relationship between the anion redox reaction, TM ion migration, and electronic structure during charging and discharging still lacks systematic and insightful research. Therefore, an in-depth understanding of the electrochemical mechanisms and constitutive relationships between the structure and performance of LRMO materials is needed, with the help of advanced characterization techniques based on synchrotron radiation and also combined with theoretical calculations; (ii) current preparation methods and modification strategies are limited in regulating the microstructure of LRMO materials and lack significant inhibition of voltage and capacity degradation. Given this, it is necessary to further innovate the synthesis method of LRMO materials and prepare LRMO materials with multi-scale regulation and a controllable microstructure; and (iii) at present, the modification strategies of most single-layer lithium-rich cathode materials are merely able to break through a single aspect of electrochemical performance. A multi-pronged and synergistic development should be attempted in the modification means, which is expected to achieve the improvement in the comprehensive electrochemical performance.

In summary, with in-depth theoretical studies and the continuous development of structural modification strategies, the application process of LRMO materials will be greatly accelerated and also breakthroughs in the technology of high-specific-energy lithium-ion batteries will be achieved. This review aims to provide summarization of some of the research progress and new modification strategies of Li-rich cathode materials, and further provide valuable suggestions and opinions for the future development of Li-rich cathode materials for LIBs with high energy density and stable durability.

## Figures and Tables

**Table 1:** Effect of different modification methods on LRMO cathode materials.

Materials	Modification	Initial (mAh g^−1^/C)	ICE (%)	Rate (mAh g^−1^/C)	Retention (%/Cycles/C)	Ref.
	Surface Coating					
Li_1.256_Ni_0.198_Co_0.082_Mn_0.689_O_2.25_	Al_2_O_3_	282.0 mAh g^−1^/0.1C	87.4%	189.0 mAh g^−1^/5C	95.2%/60/1C	[71]
Li_1.2_Mn_0.54_Ni_0.13_Co_0.13_O_2_	ZnO	271.8 mAh g^−1^/0.1C	84.3%	149.7 mAh g^−1^/5C	97.5%/100/0.5C	[72]
Li_1.2_Mn_0.54_Ni_0.13_Co_0.13_O_2_	MnO_2_	296.9 mAh g^−1^/0.05C	93.0%	165.0 mAh g^−1^/5C	92.84%/100/1C	[73]
Li_1.2_Mn_0.54_Ni_0.13_Co_0.13_O_2_	Nb_2_O_5_	258.0 mAh g^−1^/0.1C	83.4%	152.0 mAh g^−1^/5C	97.2%/100/0.5C	[74]
Li_1.2_Mn_0.54_Ni_0.13_Co_0.13_O_2_	CeO_2_	265.0 mAh g^−1^/0.1C	83.3%	158.0 mAh g^−1^/5C	94.9%/100/0.5C	[76]
Li_1.2_Mn_0.54_Ni_0.13_Co_0.13_O_2_	AlF_3_	283.3 mAh g^−1^/0.1C	88.3%		84.4%/200/1C	[78]
Li_4_Mn_5_O_12_	MgF_2_	268.9 mAh g^−1^/0.1C	93.0%	140.8 mAh g^−1^/5C	80.0%/300/0.5C	[79]
Li_1.2_Mn_0.54_Ni_0.13_Co_0.13_O_2_	LiF	242.4 mAh g^−1^/0.1C	80.0%		95.0%/100/1C	[80]
Li_1.2_Mn_0.54_Ni_0.13_Fe_0.13_O_2_	LiF-MgF_2_-CaF_2_	240.6 mAh g^−1^/0.1C	72.1%	121.2 mAh g^−1^/5C	90.1%/120/0.2C	[83]
Li_1.2_Mn_0.54_Ni_0.13_Co_0.13_O_2_	Li_3_PO_4_	273.0 mAh g^−1^/0.1C	86.9%	143.8 mAh g^−1^/5C	81.8%/175/0.5C	[85]
Li_1.08_Mn_0.54_Co_0.13_Ni_0.13_O_2_	Li_2_TiO_3_	276.5 mAh g^−1^/0.1C	86.3%		99.7%/125/0.2C	[87]
Li_1.2_Ni_0.2_Mn_0.6_O_2_	LiAlO_2_	268.8 mAh g^−1^/0.1C	84.5%	143.8 mAh g^−1^/5C	97.3%/100/0.C	[88]
Li_1.2_Mn_0.54_Ni_0.13_Co_0.13_O_2_	Li_2_MnO_3_	294.0 mAh g^−1^/0.1C	87.6%	211.1 mAh g^−1^/1C	79.6%/100/0.5C	[89]
Li_1.2_Mn_0.6_Ni_0.2_O_2_	Li_2_ZrO_3_	239.8 mAh g^−1^/0.1C		140.2 mAh g^−1^/2C	83.5%/100/1C	[90]
LiNi_x_Mn_y_Co_x_O_2_	Li_x_Si_y_O_z_	294.0 mAh g^−1^/0.1C		200.0 mAh g^−1^/4C	81.4%/200/0.3C	[91]
Li_1.2_Mn_0.54_Ni_0.13_Co_0.13_O_2_	KPB	281.7 mAh g^−1^/0.1C	85.69%	139.0 mAh g^−1^/5C	77.17%/100/0.5C	[92]
Li_1.2_Mn_0.54_Ni_0.13_Co_0.13_O_2_	LLZAO	282.4 mAh g^−1^/0.1C	85.6%	123.9 mAh g^−1^/5C	95.7%/300/1C	[93]
Li_1.2_Mn_0.54_Ni_0.13_Co_0.13_O_2_	C@spinel/MO	283.3 mAh g^−1^/0.1C	85.0%		89.7%/100/1C	[94]
	**Elemental Doping**					
Li_1.2_Ti_0.26_Ni_0.18_Co_0.18_Mn_0.18_O_2_	Ti^2+^	176.0 mAh g^−1^/0.1C	60%		97%/180/0.1C	[95]
LiNi_1/3_Mn_1/3_Co_1/3_O_2_	Zn^2+^	191.0 mAh g^−1^/0.1C	66.1%		70%/100/0.1C	[96]
Li_1.2+x_Mn_0.54_Ni_0.13_Co_0.13−x−y_Al_y_O_2_	Al^3+^	225.0 mAh g^−1^/0.1C		124.0 mAh g^−1^/2C	100%/200/0.1C	[97]
Li_1.2_Mn_0.54_Ni_0.13_Co_0.13_O_2_	Nb^5+^	287.5 mAh g^−1^/0.1C	86.94%	123.6 mAh g^−1^/8C	98.5%/300/0.2C	[98]
Li_1.2_Mn_0.54_Ni_0.13_Co_0.13_O_2_	La^3+^	262.1 mAh g^−1^/0.1C	81.5%		86.7%/50/1C	[99]
Li_1.2_Na_0.03_[Ni_0.2464_Mn_0.462_Co_0.0616_]O_2_	Na^+^	250.2 mAh g^−1^/0.1C		138.4 mAh g^−1^/5C	97.17%/110/1C	[100]
Li_1.2_Mn_0.54_Ni_0.13_Co_0.13_O_2_	K^+^	294.6 mAh g^−1^/0.1C	80.7%	110.8 mAh g^−1^/10C	93.4%/200/0.5C	[101]
Li_1.2_Mn_0.6_Ni_0.2_O_2_	Cr^6+^	200.0 mAh g^−1^/1.0C			99.0%/200/1C	[102]
Li_1.2_Mn_0.54_Ni_0.13_Co_0.13_O_2_	Ni/Mg			94.84 mAh g^−1^/7C	78.63%/300/5C	[103]
Li_1.2_Mn_0.54_Ni_0.13_Co_0.13_O_2_	S^2-^	293.3 mAh g^−1^/0.1C	96.06%	117.0 mAh g^−1^/5C	72.7%/67/1C	[106]
Li_1.2_Mn_0.54_Ni_0.13_Co_0.13_O_2_	Fe & Cl	235.1 mAh g^−1^/0.2C	73.1%	145.6 mAh g^−1^/5C	86.4%/500/1C	[111]
Li_1.2_Ni_0.2_Mn_0.6_O_2_	Na & F			135.0 mAh g^−1^/5C	100%/100/0.2C	[112]
	**Structural Designs**					
Li_1.2_Mn_0.54_Ni_0.13_Co_0.13_O_2_	multi-functionalized full-interface integrated engineering				86.6%/500/1C	[113]
Li_1.2_Mn_0.6_Ni_0.2_O_2_	layered/rock-salt intergrown structure	288.4 mAh g^−1^/0.1C		131.8 mAh g^−1^/10C	88.2%/100/0.5C	[114]
Li_1.2_Mn_0.4−x_Ti_0.4_CrxO_2_	rock-salt typeformations	253.0 mAh g^−1^/0.1C				[118]
Li_1.2_Mn_0.54_Ni_0.13_Co_0.13_O_2_	single crystal	260.0 mAh g^−1^/0.1C			97.6%/100/0.1C	[119]
	**High Entropy**					
Li_1.0_[Li_0.1_5Mn_0.50_Ni_0.15_Co_0.10_Fe_0.025_Cu_0.025_Al_0.025_Mg_0.025_]O_2_	Fe, Cu, Al, Mg	260.0 mAh g^−1^/0.1C	85%		93.4%/100/0.1C	[124]
LiNi_0.8_Mn_0.13_Ti_0.02_Mg_0.02_Nb_0.0_1Mo_0.02_O_2_	Ti, Mg, Nb, Mo	210.1 mAh g^−1^/0.1C	94%		95%/500/1C	[125]

## Data Availability

No data were used for the research described in the article.
